# Klippel-Trenaunay syndrome presenting with acanthocytosis and splenic and retroperitoneal lymphangioma: a case report

**DOI:** 10.1186/1752-1947-8-390

**Published:** 2014-11-27

**Authors:** Milinda Withana, Chaturaka Rodrigo, Mitrakrishnan Chrishan Shivanthan, Sachini Warnakulasooriya, Manu Wimalachandra, Lallindra Gooneratne, Senaka Rajapakse

**Affiliations:** 12 University Medical Unit, National Hospital, Colombo, 10 CO008 Sri Lanka; 13 Department of Clinical Medicine, Faculty of Medicine, University of Colombo, 25, Kynsey Road, Colombo 08, Sri Lanka; 14 Department of Pathology, Faculty of Medicine, University of Colombo, 25, Kynsey Road, Colombo 08, Sri Lanka

## Abstract

**Introduction:**

Klippel-Trenaunay syndrome is a rare congenital mesodermal abnormality characterized by bone and soft tissue hypertrophy, extensive hemangioma and venous abnormalities. We report the case of a patient with two additional rare clinical manifestations in the background of Klippel-Trenaunay syndrome, namely, acanthocytosis and splenic and retroperitoneal lymphangioma.

**Case presentation:**

A 24-year-old Sri Lankan man from North Central Province in Sri Lanka presented to our general medical unit with symptomatic anaemia. He had been diagnosed with Klippel-Trenaunay syndrome at the age of six years, with hemihypertrophy of his right lower limb and strawberry naevi over both lower limbs. His blood film results were positive for acanthocytes, which accounted for more than 20% of the red blood cell population. He was also found to have extensive splenic lymphangiomas and a large retroperitoneal lymphangioma encasing the mesentric vessels in the right para-aortic region. An extensive battery of tests to identify a secondary cause for the acanthocytosis failed to show any positive results.

**Conclusions:**

Retroperitoneal lymphangioma has been reported in association with Klippel-Trenaunay syndrome once before, but an association with acanthocytosis has never been reported. Given the rarity of all three conditions this is not surprising. The cause of acanthocytosis in this setting is currently unresolved. It is plausible that this may be a primary association with Klippel-Trenaunay syndrome, as an alternative aetiology was not found.

## Introduction

Klippel-Trenaunay syndrome is a congenital mesodermal anomaly characterized by the triad of varicose veins, cutaneous capillary malformations and hypertrophy of bone and/or soft tissue [1].

Acanthocytosis refers to the transformation of an erythrocyte’s normal biconcave disc shape into a cell with several irregularly shaped external projections distributed unevenly on the membrane surface [2]. This is associated with a variety of inherited and acquired disorders. To the best of our knowledge, acanthocytosis in association with Klippel-Trenaunay syndrome has not been reported in the medical literature so far.

Retroperitoneal lymphangioma is a rare congenital malformation arising from the retroperitoneal lymphatics. Only 5% of lymphangioma are seen in the abdomen and a retroperitoneal location is exceedingly rare [3]. Klippel-Trenaunay syndrome associated with retroperitoneal lymphangioma has been reported only once in the medical literature [3].

We report the case of a patient with Klippel-Trenaunay syndrome who also had extensive lower gastrointestinal varices, skin hemangiomas, retroperitoneal and splenic lymphangioma and acanthocytosis.

## Case presentation

A 24-year-old Sri Lankanman from North Central Province in Sri Lanka presented to our general medical unit with symptomatic anemia. He was diagnosed with Klippel-Trenaunay syndrome at the age of six years, with hemihypertrophy of his right lower limb and strawberry naevi over both lower limbs. He did not have any additional symptoms until 17 years of age when he experienced episodic bleeding of the rectum. A colonoscopy and mesenteric angiogram performed at that time revealed lower gastrointestinal varices involving the colon and rectum.

**Figure 1:**
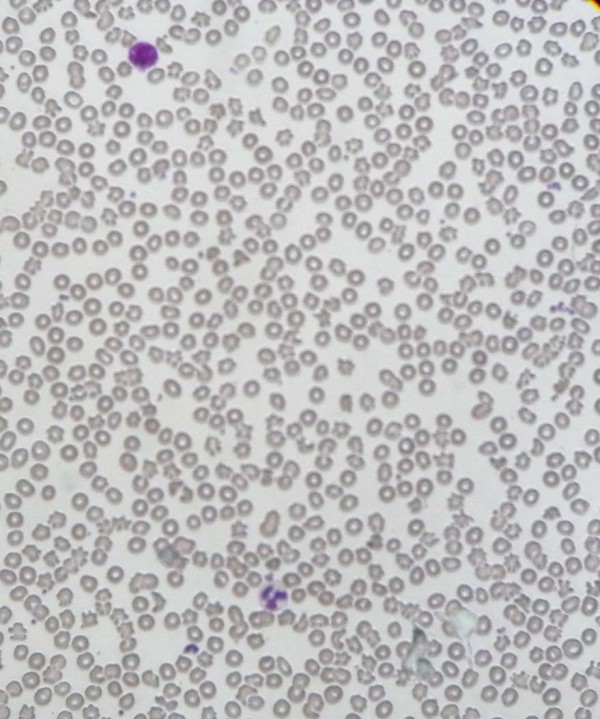
**Peripheral blood film of the patient showing acanthocytosis accounting for more than 20% of the red blood cell population.**

**Figure 2:**
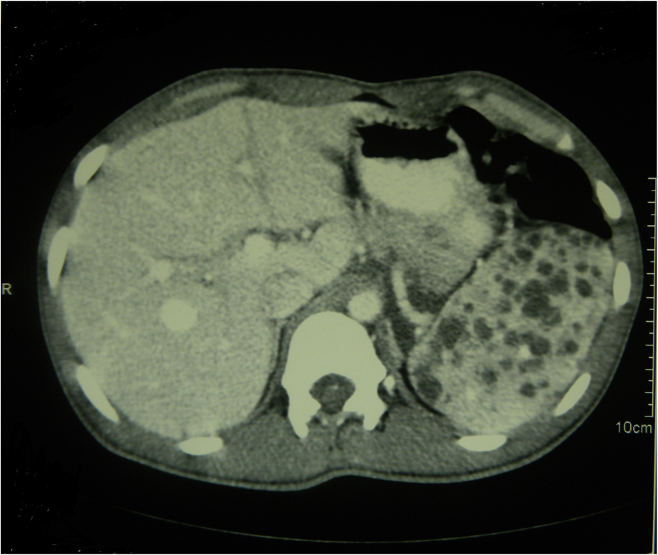
**Contrast-enhanced computed tomography scan of the abdomen showing multiple cystic lesions in the spleen suggestive of splenic lymphangioma.**

**Figure 3:**
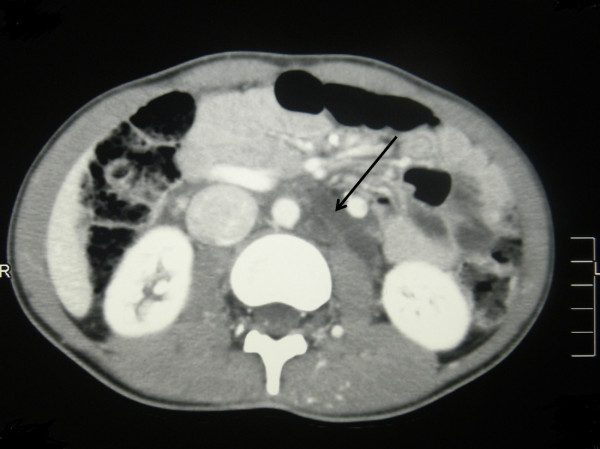
**Contrast enhanced computed tomography of the abdomen showing the retroperitoneal lymphangioma (see arrow).**

He presented to our department with effort intolerance (New York Heart Association classification stage three) and lethargy. The clinical picture was not suggestive of a respiratory cause. Upon his examination, other than the features of Klippel-Trenaunay syndrome, pallor with splenomegaly was noted. His spleen was palpable 1cm below the costal margin. There was no hepatomegaly or lymphadenopathy. There were no neurological abnormalities such as deafness, cerebellar signs, peripheral neuropathy or retinitis pigmentosa.Laboratory investigations revealed a hemoglobin level of 8.9g/dL with the following red blood cell indices: a mean corpuscular volume of 77.6μL, hematocrit at 28%, a mean corpuscular hemoglobin concentration of of 31.8g/dL and a mean corpuscular hemoglobin level of 24.7pg. His white cell count and platelet count were within reference ranges. A blood picture showed a mixed population of hypochromic microcytic and normochromic red blood cells. There was significant acanthocytosis exceeding 20% of all red blood cells (Figure 1) and his reticulocyte count was 4%. A direct Coombs’ test was negative. The results of other biochemical investigations are as follows: a serum lactate dehydrogenase (LDH) level of 360U/L (230 to 460), an aspartate aminotransferase (AST) level of 34U/L (10 to 35), an alanine aminotransferase (ALT) level of 36U/L (10 to 40), an alkaline phosphatase (ALP) level of 199U/L (100 to 360), a serum total bilirubin level of 16umol/L (5 to 21), a direct bilirubin level of 3.1umol/L (<3.4), a total protein level of 69g/L (61 to 77), an albumin level of 40g/L (36 to 48), a serum sodium level of 144mmol/L (135 to 148), a serum potassium level of 4.1mmol/L (3.5 to 5.3), a serum creatinine level of 61umol/L(60 to 120), a serum ionized calcium level of 1.2mmol/L(1 to 1.3), a magnesium level of 0.9mmol/L (0.8 to 1.1), a serum iron level of 22ug/dL (37 to 148), a total iron binding capacity of 256ug/dL (274 to 386) and transferrin saturation at 8.5% (15 to 50). His thyroid hormone profile was within normal limits and his lipid profile did not show any marked abnormalities; total cholesterol of 173mg/dL (<240), low density lipoprotein (LDL) cholesterol of 103mg/dL (<160), high density lipoprotein (HDL) cholesterol of 49mg/dL (>45), triglycerides at 102mg/dL (<150) and very low density lipoproteins (VLDL) cholesterol of 20.4mg/dL (<40). Blood pictures of first degree family members including his mother, brother and sister did not show acanthocytosis. A colonoscopy at a previous hospital admission four months ago showed extensive hemangioma in transverse and descending colon plus rectal varices.An ultrasound scan of his abdomen revealed a splenomegaly of 15cm with multiple cystic lesions. A triple phase contrast-enhanced computed tomography (CT) scan of his abdomen and pelvis showed multiple cystic lesions in the spleen (Figure 2) without any contrast enhancement at any phase of the scan. There was also a diffuse hypodense (-4 Hounsfield units) nonenhancing retroperitoneal lesion in his right para-aortic region encasing the mesentric vessels. The appearance of this lesion and those in his spleen were suggestive of multiple lymphangioma (Figure 3).

A transthoracic two-dimensional echocardiogram showed a left ventricular ejection fraction of 60%, a left ventricular cardiac output of 3.3L/min with a cardiac index of 2.3L/min/m^2^. This ruled out high output cardiac failure as a cause of his symptoms.

His breathlessness was diagnosed as iron deficiency due to intermittent rectal bleeding. He was treated with oral iron for three months, resulting in the resolution of symptoms. While his hemoglobin level improved to 11g/dL, a blood film revealed that acanthocytes were still accounting for more than 20% of his red blood cells. The gastroenterology team offered a partial proctocolectomy but he did not consent to the surgery. He is currently symptomatically managed with iron supplements and is regularly followed up on a monthly basis in the clinic.

## Discussion

In 1900, Klippel and Trenaunay described a rare congenital disorder of mesodermal development, manifesting as dermal capillary malformation (usually a port wine stain over the affected limb or at a different site), soft-tissue and/or bone hypertrophy and varicose veins or venous malformation, sometimes with persistent lateral embryonic veins [4]. Of these symptoms, our patient had a hypertrophied lower limb, multiple strawberry naevi in the bilateral lower extremities and hemangioma of the colon.

The hypodense non-enhancing retroperitoneal lesion in the contrast-enhanced CT scan was suggestive of a lymphangioma. Lymphangiomas are benign malformations analogous to hemangiomas composed of endothelium-lined cystic spaces, containing proteinaceous fluid. However, the retroperitoneal location is rare and occurs in less than 5% of instances. It has been described in association with Klippel-Trenaunay syndrome once before [3]. Most lymphangiomas occur in childhood, and they frequently accompany diffuse lymphoid angiomatosis. CT scans show hypodense, non-enhancing discrete areas, with sharp, thin margins [5]. The appearance of splenic lesions in the contrast-enhanced CT scan of our patient was also compatible with lymphangiomas.

Acanthocytosis denotes transformation of normal biconcave shaped red blood cell membrane into one with unevenly distributed external projections on its outer surface [2]. Acanthocytosis has been reported in association with several hereditary and acquired conditions, including McLeod phenotype, In(Lu) phenotype, hereditary spherocytosis with a β-spectrin deficiency, alcoholic cirrhosis, uremia, vitamin E deficiency, anorexia nervosa and hypothyroidism [2]. To the best of our knowledge it has never been reported in association with Klippel-Trenaunay syndrome. Therefore we had to extensively investigate our patient and his family members for other plausible differential diagnoses for acanthocytosis. Neither our patient nor his family members had an alternative diagnoses to explain the acanthocytosis.

In view of this we had to search for an alternative hypothesis which would explain its co-occurrence with Klippel-Trenaunay syndrome or its associations. One possible explanation was that the extensive lesions in his spleen could have affected the red blood cell morphology. However, splenic lymphangiomas have not been associated with acanthocytosis before and neither have splenic hemangiomas, which are a more common vascular lesion of the spleen [6]. Kasabach-Merritt syndrome, which is associated with giant hemangiomas, typically produces a thrombocytopenia but not morphological abnormalities in red blood cells [6]. However, it is interesting to note that sometimes a splenectomy is offered as a treatment for acanthocytosis, and the percentage of acanthocytes markedly decrease after splenectomy [6]. Therefore, we suspect that his splenic lesions may have a role to play in the aetiology of acanthocytosis. Klippel-Trenaunay syndrome is a rare entity and some of its rare associations have been observed on only a few occasions in patients. Reporting of the potential association of extensive splenic and retroperitoneal lymphangiomas and gastrointestinal hemangiomas with acanthocytosis in the background of Klippel-Trenaunay syndrome will alert other clinicians to the possible diagnosis and to report cases of similar patients. If this observation is repeatedly encountered then it can be established as a recognized association and may avoid unnecessary testing to find a secondary cause for the acanthocytosis in such patients. Different characteristics and co-existent pathologies of such patients will also help to pinpoint the cause of acanthocytosis in the background of Klippel-Trenaunay syndrome.

## Conclusions

Our patient with Klippel-Trenaunay syndrome also had two unusual co-existent manifestations of extensive lymphangioma and acanthocytosis. Since, to the best of our knowledge, acanthocytosis has never before been reported in Klippel-Trenaunay syndrome, we performed an extensive battery of tests on our patient and his first degree relatives which failed to yield a cause for the abnormal red blood cell morphology. Since Klippel-Trenaunay syndrome is an abnormality in mesodermal development and removal of the spleen is sometimes known to help in cases of extensive acanthocytosis, either Klippel-Trenaunay syndrome itself or the associated splenic lymphangiomas may have a role to play in the genesis of acanthocytosis in this setting.

## Consent

Written informed consent was obtained from the patient for publication of this case report and accompanying images. A copy of the written consent is available for review by the Editor-in-Chief of this journal.

## Authors’ information

MW (MBBS) is a registrar in medicine attached to the University Medical Unit of National Hospital of Sri Lanka. CR (MBBS, MD) is a lecturer in medicine at the Department of Clinical Medicine, Faculty of Medicine, University of Colombo. MWL (MBBS) and SW (MBBS) are registrars in hematology at the National Hospital of Sri Lanka. MCS (MBBS, MD, MRCP) is a senior registrar of the University Medical Unit. LG (MBBS, MD, FRCPath) is a consultant hematologist and senior lecturer attached to the Department of Pathology, Faculty of Medicine, University of Colombo. SR (MD, FRCP, FRCPE, FACP, FCCP) is a professor in medicine at the Department of Clinical Medicine, Faculty of Medicine, University of Colombo.

## Abbreviations

LDH: Lactate dehydrogenase
AST: Aspartate aminotransferase
ALT: Alanine aminotransferase
ALP: Alkaline phosphatase
LDL: Low density lipoprotein
HDL: High density lipoprotein
VLDL: Low density lipoprotein
CT: Computed Tomography.
